# Society Work

**Published:** 1893-05

**Authors:** 


### Society Work.

The following from *The Boston Medical and Surgical Journal* applies so well to the Dental Profession, as well as the Medical, that we venture its reproduction here.

The dental profession to a certain extent is well organized, almost every State having its society, and in nearly every State there well be found more or less local societies, and in nearly every city in the country of fifty thousand and more will be found a dental society, some of these, at least, in a flourishing condition and doing good work. Perhaps the need is not so much for more societies as that greater interest should be taken in their work by those who are members, and a far greater number be brought into membership, and actively engaged in the work.

Of the eighteen thousand dentists in the country there are not two thousand of these engaged in association work nor even connected with any dental society. If the proper interest in association work existed in the profession there would be at least one half the whole number of dentists in the country connected with dental societies. There is, however, a constant improvement being made in this respect, and college training is having much to do with this matter; in nearly every college will there be found the students' dental society. Here an interest will be aroused that promises much for the future. Association work has been one of the great instrumentalities for the development and growth of dental art and science.—Ed. Reg.

Interest in Medical Meetings.—How can the meetings of medical societies be made interesting? This question worries the chairman and secretary of most societies; but the non-official members generally merely criticise, or stay away, if the meetings are tiresome. The drawing power of society often depends as much upon the by-laws, and the activity of the officers as upon the capability of the members. It is necessary first to attract an audience, and then to keep them interested; this being accomplished, the value of the proceedings may be left to the individual speakers. The greatest bore is a paper which is ten times as long as it need be.

The adoption of a "Ten-Minute Paper," rule has recently become a marked feature in the work of the New York Academy of Medicine, especially in the Section in Pædiatrics, and has resulted in a marked increase in the attendance at the meetings, and a large number of concise and interesting papers. The instructions to writers of papers by the chairman contain the following: "Hippocrates and Galen may be passed with very slight notice, as they have been dead for some time and their opinions are somewhat obsolete. Scratch out the formal introduction and condense the body of the paper. End the paper where the subject matter ends, making its action like that of a piston syringe—begin, spatter, stop."

A busy man is more easily persuaded to write an informal short paper than a formal long one. The formality itself, and the necessity of clothing the main idea is often the bugbear which prompts a man to decline to write. If his contribution is to be one of the *piece de resistance* it is easier for him, and generally much easier for the audience. In drawing deductions from a case, or a series of cases, a reader is readily excused from going into the details of his process; he is not proving a geometrical problem, or if he is, his hearers had rather see the steps in print, where they can be skipped, than listen to them.

The discussion is generally an interesting part of a meeting. There is usually too little rather than too much, but it would be acceptable to most societies if those gentlemen who have a rambling and procrastinating tendency, could be helped along.

The reader as well as the listener will be more easily attracted by a condensed than by a well padded paper. No one, except perhaps the writer, thinks that there is more dignity in a long than in a short article. The reader has the advantage over the hearer in that he can skip, and look to the end for the conclusion, but the process is not a pleasant one to him. He is repelled by tables and by copies of hospital records, and attracted by short articles or well-defined descriptions of single cases.

